# Pulmonary Enteric Adenocarcinoma in a Super‐Elderly Patient: A Case Report and Literature Review

**DOI:** 10.1002/rcr2.70127

**Published:** 2025-02-19

**Authors:** Akira Shimamoto, Natsumi Kojo, Haruko Saiki, Akinobu Hayashi, Motoshi Takao

**Affiliations:** ^1^ Department of Thoracic and Cardiovascular Surgery, Department of Respiratory Medicine, and Department of Oncologic Pathology Mie University Graduate School of Medicine Tsu Japan

**Keywords:** adenocarcinoma, immunohistochemistry, pulmonary enteric adenocarcinoma (PEAC), super‐elderly patient, surgical resection

## Abstract

Pulmonary enteric adenocarcinoma (PEAC) is an extremely rare subtype of non‐small cell lung cancer, characterised by histopathological features that resemble colorectal adenocarcinoma. Here, we report a case of PEAC in an 85‐year‐old male who presented with a pulmonary nodule initially identified at the age of 76. Initial PET‐CT (maximum standardised uptake value [SUVmax] = 2.8) was inconclusive, leading to strict follow‐up. Over 9 years, the nodule enlarged significantly (3.2 cm, SUVmax = 8.4). A transbronchial biopsy confirmed adenocarcinoma, and the patient underwent successfully video‐assisted thoracoscopic surgery with lymph node dissection. The histopathological analysis revealed CK7 and CK20 positivity, focal CDX2 positivity, and TTF‐1 negativity, confirming PEAC. Despite advanced age, the patient tolerated surgery well without adjuvant therapy. At the one‐year postoperative follow‐up, he remained disease‐free. This case emphasises the diagnostic utility of immunohistochemistry and the feasibility of surgical management, even in super‐elderly patients with PEAC.

## Graphical abstract

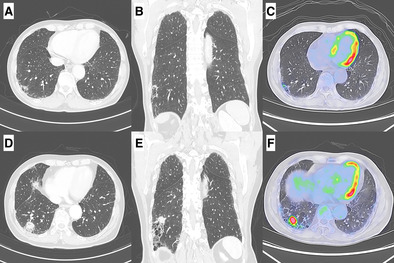

## 1 Introduction

Pulmonary enteric adenocarcinoma (PEAC) is an exceptionally rare subtype of non‐small cell lung cancer (NSCLC), accounting for less than 0.5% of all primary lung adenocarcinomas [1]. It is characterised by histopathological features resembling colorectal adenocarcinoma, including irregular glandular structures lined by columnar epithelial cells and immunohistochemical positivity for intestinal markers such as CK20 and CDX2. First described in 1991, PEAC was officially classified as a distinct variant of invasive lung adenocarcinoma by the World Health Organization (WHO) in 2015 [2].

Diagnosing PEAC is particularly challenging due to its histopathological and immunohistochemical similarities with metastatic colorectal cancer (MCRC). Accurate differentiation relies on a combination of clinical, radiological, and pathological analyses. Immunohistochemical studies play a pivotal role, especially in distinguishing PEAC from MCRC in patients without a known history of colorectal cancer.

Here, we report a rare case of PEAC in an 85‐year‐old male, emphasising the diagnostic approach, surgical treatment, and favourable postoperative outcome. This case underscores the importance of immunohistochemical markers in establishing the diagnosis and highlights the potential for successful surgical management in a super‐elderly patient.

## 2 Case Report

A male patient with a history of non‐alcoholic liver disease underwent routine whole‐body CT at the age of 76, which revealed a 5 mm solitary nodule in the lateral basal segment of the right lung. A subsequent PET‐CT was performed for further evaluation, revealing a maximum standardised uptake value (SUVmax) of 2.8. This finding made it challenging to differentiate between lung cancer and inflammation. The lesion was closely monitored through serial CT scans due to its indeterminate nature.

**FIGURE 1:**
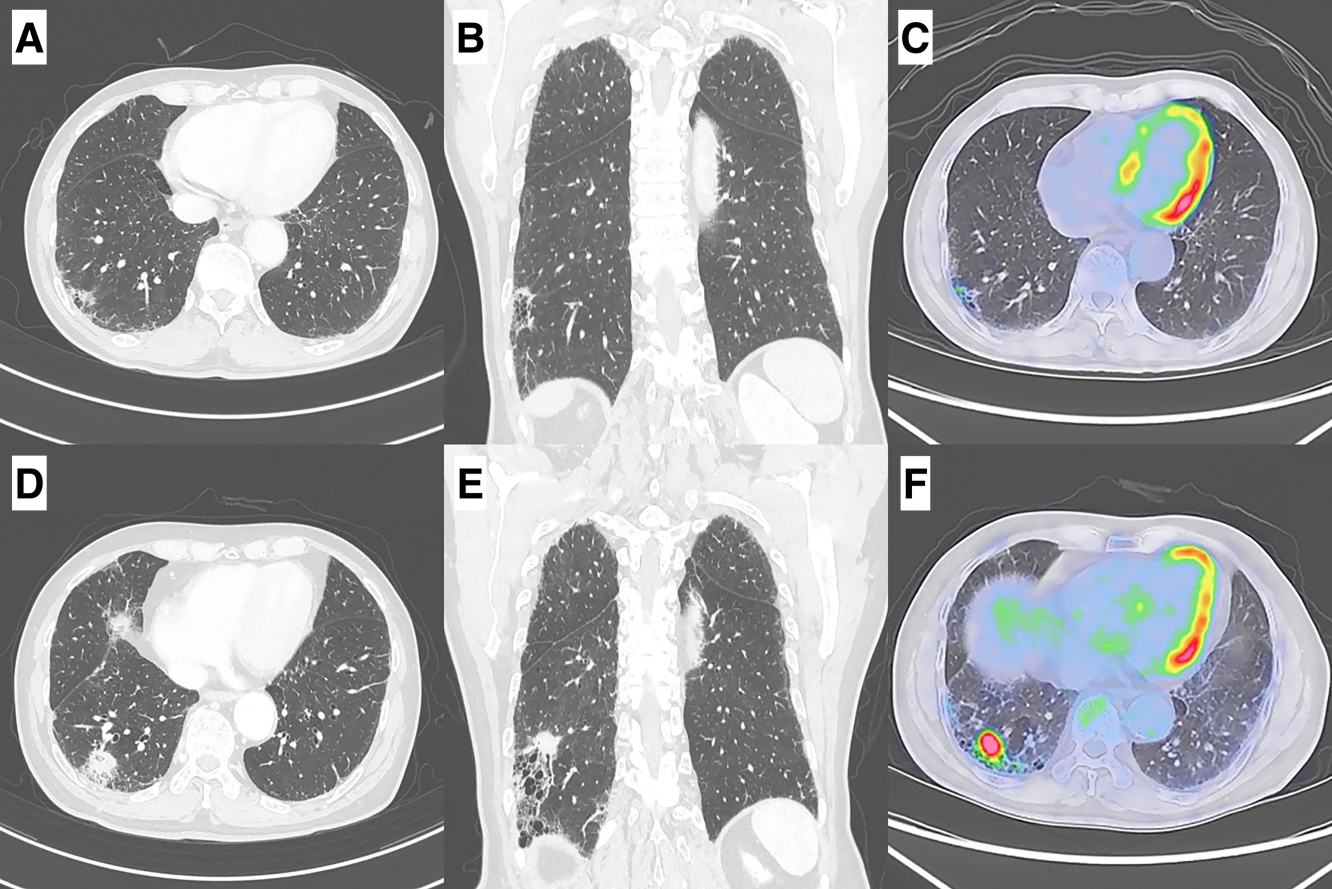
Chest CT and PET/CT findings. (A, B) Chest CT showed a 5 mm solitary nodule in the lateral basal segment of right lung at 9 years before surgery. (C) PET/CT showed an increased ^18^F‐fluorodeoxyglucose (FDG) uptake in the lesion (maximum standardised uptake value [SUVmax] = 2.8) at 9 years before surgery. (D, E) Chest CT revealed the lesion had grown to 3.2 cm immediately before surgery. (F) PET/CT revealed an increased FDG uptake (SUVmax = 8.4) in the lesion immediately before surgery.

At the age of 85, after 9 years of strict CT follow‐up, the nodule demonstrated significant enlargement, forming a solid, irregular mass measuring 3.2 cm. PET‐CT revealed significant ^18^F‐fluorodeoxyglucose uptake (SUVmax = 8.4) within the lesion, with no evidence of extrathoracic metastasis (Figure 1). A transbronchial lung biopsy confirmed the diagnosis of adenocarcinoma. Although super‐elderly, his good performance status and the localised stage of the tumour (cT2aN0M0, stage IB) made him a candidate for surgical resection. The patient also underwent upper gastrointestinal endoscopy (UGIF) and colonoscopy (CF) as part of an unrelated evaluation, revealing no abnormalities.

The patient subsequently underwent video‐assisted thoracoscopic surgery (VATS) with right lower lobectomy and systematic lymph node dissection (ND2a‐1). The postoperative course was uneventful, with the chest drain removed on postoperative day 1 and discharge on postoperative day 4.

**FIGURE 2:**
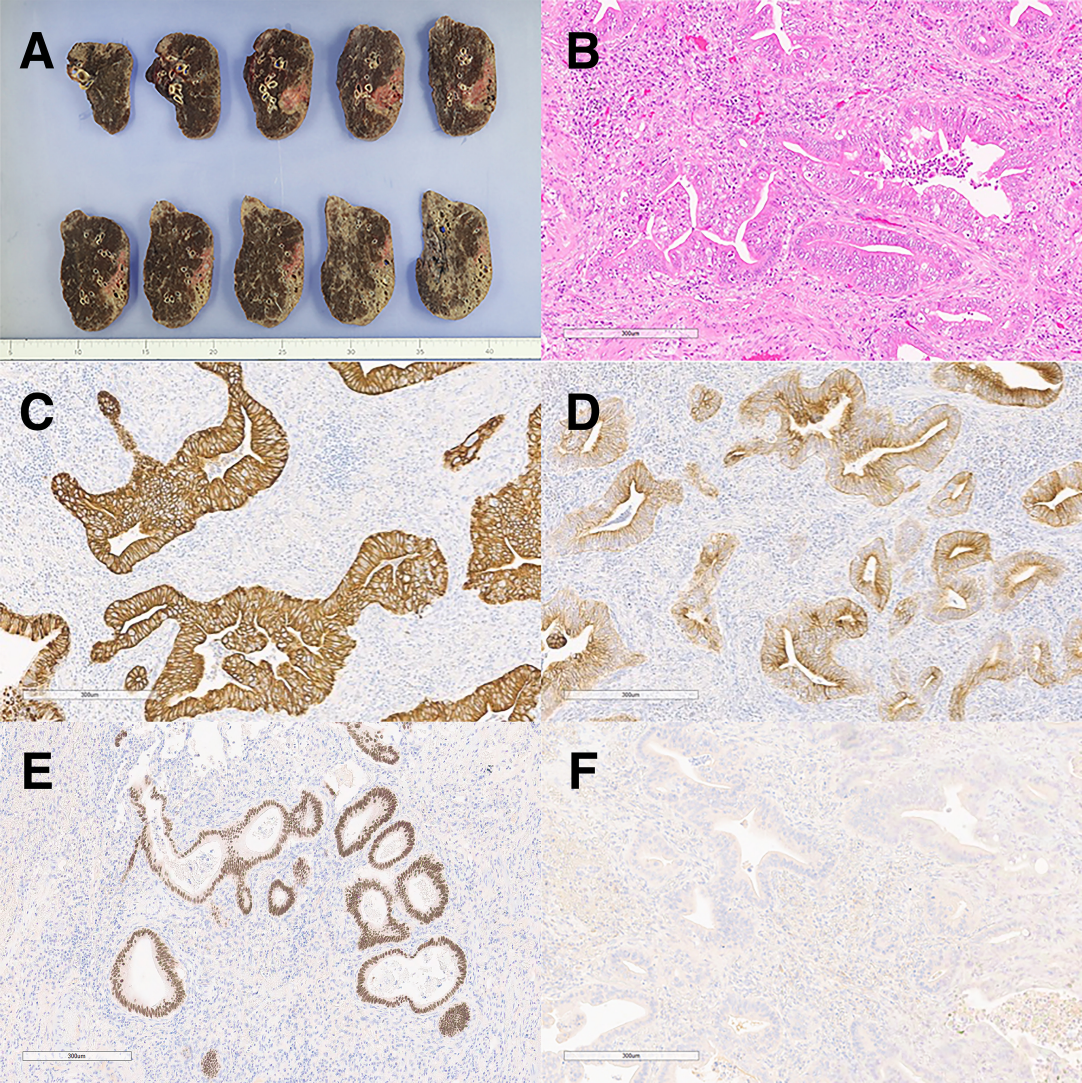
Representative histopathological findings of the resected specimen. (A) Macroscopic image of the resected specimen showing a well‐demarcated tumour in the lateral basal segment of the right lung. (B) Haematoxylin and eosin staining demonstrating irregular glandular structures with columnar epithelial cells exhibiting eosinophilic cytoplasm. (C) Immunohistochemical staining showing strong positivity for CK7. (D) Immunohistochemical staining showing positivity for CK20. (E) Immunohistochemical staining showing focal positivity for CDX2. (F) Immunohistochemical staining showing negativity for TTF‐1.

Detailed histopathological examination of the resected specimen revealed irregular glandular structures with columnar epithelial cells exhibiting eosinophilic cytoplasm. Immunohistochemical analysis showed positivity for CK7 and CK20, focal positivity for CDX2, and negativity for TTF‐1 and MUC‐2 (Figure 2). These findings confirmed the diagnosis of PEAC. The tumour, including its invasive component, measured 4.2 cm in maximum diameter upon histopathological examination, resulting in a final pathological stage of pT2bN0M0 (stage IIA).

Considering his advanced age and lack of high‐risk features, adjuvant therapy was not administered. At the one‐year postoperative follow‐up, the patient remained disease‐free with no evidence of recurrence.

## 3 Discussion

PEAC is a diagnostically challenging and rare subtype of NSCLC. First reported in 1991 and officially classified by the WHO in 2015, PEAC accounts for less than 0.5% of all primary lung adenocarcinomas. To date, fewer than 100 cases have been documented in the literature. The rarity of PEAC has resulted in a lack of standardised diagnostic and treatment guidelines, and most published studies consist of single‐institution case series or isolated case reports.

Accurate differentiation between PEAC and MCRC is critical due to their overlapping histopathological and immunohistochemical characteristics. Key immunohistochemical markers, including CK20, CDX2, and TTF‐1, play a pivotal role in distinguishing PEAC from other adenocarcinomas. CK20 and CDX2 positivity, combined with TTF‐1 negativity, strongly suggest intestinal differentiation in primary lung adenocarcinomas. Recent studies have also emphasised the utility of next‐generation sequencing alongside immunohistochemistry in differentiating PEAC from MCRC [3]. Additionally, the absence of gastrointestinal symptoms, no history of colorectal cancer, and normal findings on UGIF and CF examinations were critical in ruling out a primary gastrointestinal malignancy.

Surgical resection remains the cornerstone of treatment for localised PEAC, as supported by multiple case studies. In elderly patients, the risks and benefits of surgery must be carefully weighed. Despite his advanced age, the patient successfully underwent VATS with systematic lymph node dissection, demonstrating that advanced age should not preclude surgical consideration in well‐selected cases. The procedure was well tolerated, and the postoperative course was uneventful, highlighting the feasibility of surgical management even in super‐elderly patients with good performance status.

The prognosis of PEAC appears comparable to that of conventional NSCLC when appropriately treated [4]. Emerging studies highlight that PEAC may harbour actionable mutations, including KRAS and mismatch repair deficiencies, suggesting potential avenues for personalised therapy and future clinical trials [5]. Although our patient did not receive adjuvant therapy due to his advanced age and early‐stage disease, the absence of recurrence at the one‐year follow‐up supports the efficacy of surgical intervention alone in this context.

This case is consistent with existing literature in terms of immunohistochemical profile and favourable surgical outcomes. However, it underscores the importance of individualised surgical planning and careful risk assessment, particularly in super‐elderly patients. Further research is needed to establish the role of targeted therapies and immunotherapy in the management of PEAC and to develop standardised diagnostic and treatment protocols.

## Author Contributions

Akira Shimamoto wrote the manuscript. Haruko Saiki examined the patient, and Akira Shimamoto, Natsumi Kojo, and Motoshi Takao performed surgery. Akinobu Hayashi diagnosed pathologically. All authors have approved the final version of the manuscript for submission.

## Ethics Statement

The authors declare that appropriate written informed consent was obtained for the publication of this manuscript and accompanying images.

## Conflicts of Interest

The authors declare no conflicts of interest.

## Data Availability

The data that support the findings of this study are available on request from the corresponding author. The data are not publicly available due to privacy or ethical restrictions.
